# A political economy analysis of strengthening health information system in Tanzania

**DOI:** 10.1186/s12911-023-02319-9

**Published:** 2023-10-30

**Authors:** August Kuwawenaruwa, Henry Mollel, John Matiko Machonchoryo, Federica Margini, Jennie Jaribu, Peter Binyaruka

**Affiliations:** 1 Ifakara Health Institute, Plot 463, Kiko Avenue Mikocheni, P.O. Box 78 373, Dar es Salaam, Tanzania; 2 Mzumbe University, P.O Box 1, Mzumbe, Morogoro, Tanzania; 3 UNICEF, P.O. Box 4076, Dar es Salaam, Tanzania

**Keywords:** Health Information System, HMIS, Integration, Interoperability, Tanzania

## Abstract

**Background:**

Many countries’ health systems are implementing reforms to improve the functioning and performance of the Health Management Information System (HMIS) to facilitate evidence-based decisions for delivery of accessible and quality health services. However, in some countries such efforts and initiatives have led to a complex HMIS ecosystem characterized by multiple and fragmented sub-systems. We undertook an in-depth analysis of the HMIS ecosystem in Tanzania to inform the ongoing initiatives, by understanding the relationship and power differences among stakeholders, as well as drivers and barriers to HMIS investment and strengthening.

**Methodology:**

This was a qualitative research method incorporating data collection through document review and key informant interviews guided by political economy analytical framework. A total of 17 key informant interviews were conducted between April and May 2022. A thematic content analysis was used during data analysis.

**Results:**

Good relationship between the government and stakeholders dealing/supporting HMIS ecosystem was noted as there are technical working groups which brings stakeholders together to discuss and harmonize HMIS activities. The ‘*need for the data*’ has been the driving force toward investment in the HMIS ecosystem. The analysis showed that the government is the main stakeholder within the HMIS ecosystem and responsible for identifying the needs for improvement and has the power to approve or reject systems which are not in line with the government priority as stipulated with the HMIS investment roadmap/strategy. Moreover, partners with long relationship are powerful in influencing HMIS investment decision-making compared to those who are recently coming to support. It was further noted shortage of staff with technical competence, inadequate financial resources, and the development of fact that some of the existing systems have not been developed to their full capacity and have hindered the whole systems’ integration and interoperability exercise of ensuring integration and interoperability of the systems.

**Conclusion:**

A need-based assessment of staff capacity at the sub-national level is equally important to identify available capabilities and the knowledge gap to strengthen the HMIS ecosystem. Strong coordination of the ideas and resources intended to strengthen the HMIS ecosystem would help to reduce fragmentation. In addition, there is a need to mobilize resources within and outside the country to facilitate the integration and interoperability process smoothly.

**Supplementary Information:**

The online version contains supplementary material available at 10.1186/s12911-023-02319-9.

## Background

Strengthening the health information system is important and relevant in many countries [1]. Health information are critical to inform evidence-based decision making [2]. However, in some countries like Tanzania, the use of health information for decision-making has been limited, and there are several parallel and uncoordinated systems for data collection designed for specific needs. This creates the need for integration to integrate the existing programs and systems into a broader health-sector data warehouse as a central source of health information [3]. The Tanzanian health sector strategic plan (HSSP) IV and V include strategic directions around strengthening the health management information system (HMIS) to improve data use [4, 5]. These include; embracing the rapid development of Information and Communication Technology (ICT) for systems strengthening and stimulating the development and guiding the integration and interoperability of systems; and secondly, having Monitoring and Evaluation systems focus on data-for-decision making, utilising web-based data collection and analysis, linking information systems, and providing stakeholders access to data.

The HMIS ecosystem in Tanzania has multiple and fragmented systems [4, 6] and limited interoperability. Despite many initiatives undertaken, most of the health information systems operate in silos with specific reporting purposes (e.g., vertical programmes), and end up overburdening frontline providers at the lower level. Currently, many developing countries, including Tanzania, recognize the importance of digital health technology, but its adoption and implementation continue to be limited by several persistent challenges or constraints. Inadequate human and financial resources, lack of incentives and proper supervision, unreliable electricity supply, intermittent internet connectivity and absence of standard operating procedures on data management has prevented HMIS from achieving its full potential not only in Tanzania [7, 8] but also across Africa [9, 10]. Digital systems simplify the collection and analysis of data and improve the accuracy, completeness, reliability and timeliness of data used for decision-making. Digital systems need technical interoperability standards, transparent governance and legal protections for all patient data. Data confidentiality and security are particularly important for key populations including vulnerable population and people living with chronic conditions [11]. The government of Tanzania’s digital health strategy (2019–2024) seeks to provide a strategic direction in the development, adaptation, harmonization, integration and deployment of digital health solutions to ultimately improve the effectiveness and efficiency of delivering health care services [12]. Improved HMIS in the country would enhance evidence-based decision and policy-making, hence leading to improved accountability and effectiveness at all health system levels from national to sub-national levels [7].

To improve the optimization of the HMIS, one would need to explore in detail the processes and inputs required to strengthen HMIS in Tanzania. In addition, understanding the relationship between various stakeholders in influencing the design, adoption, financing, and implementation of the HMIS in Tanzania will be vital. Considering the above background, a political economy analysis was conducted to explore in detail the status of the HMIS ecosystem in Tanzania. The main objective of the study was to inform the ongoing initiatives, understand the relationship among various stakeholders and recommend strategies for improvement of the HMIS ecosystem using the political economy analysis framework.

## Methods

### Study setting

In 1993, the government of Tanzania decentralized and devolved administrative and fiscal management to the district level in various sectors including health [13, 14]. The aim was to improve planning and allocation of resources to meet various sub-national needs. This stimulated the need for reliable health information systems for data-use in planning and decision-making. The government of Tanzania, in collaboration with development partners, piloted a system in Mbeya before nationally scaling up the semi-computerised Health Information System version 1 between 1994 and 1997 [15]. The rationale of introducing HMIS was to optimize the performance of health services at all levels of administration by providing necessary and sufficient information needed by the health system managers to monitor, evaluate, and plan their activities. Since the first version of HMIS was in English, there was a need to develop a version 2 in 1998 in Swahili and make other adjustments to ensure user-friendliness. Despite the introduction of the HMIS, data collection at facility level continued to be done manually with monthly tabulations imported in the 12 HMIS booklets including forms and registers [15]. These booklets included forms and registers. The introduction of HMIS was perceived as promising, but several challenges prevailed including the lack of trust in the data reported due to unreliability [16].

To date, Tanzania has implemented a couple of changes in a bid to improve the health information system in the country and ensure the friendly use of data for evidence-based planning and decisions. These include the upgrading of the HMIS to a partially computerized system using the District Health Information System version 2 (DHIS2) software, and the consolidation of some vertical programme data into the main HMIS (e.g., HIV, TB, malaria) [5]. In 2010, Tanzania introduced the Monitoring and Evaluation (M&E) Strengthening Initiative (MESI 2010–2015) to review and update the new paper-based HMIS and the introduction of the second version of the District Health Management Information System (DHIS2) [17]. The DHIS2 is a web-based software package for collecting, validation, and analysis of data. The DHIS2 was piloted and eventually rolled out to all district councils in Tanzania in 2013. Currently, more than 90% of data flows from health facilities to higher levels through DHIS2 in Tanzania [5]. Apart from the DHIS2 platform for service utilization data, Tanzania also has other health information systems (see Table 1). Further efforts are being made to reduce the fragmentation of health information systems by integrating the DHIS2 with other systems like the Health Facility Registry (HFR) and the Human Resource for Health Information System (HRHIS). However, there is still a need to further integrate and ensure interoperability for easy data exchange between various health information systems in Tanzania.

**Table 1:** Health Information System Reforms in Tanzania

	Health Information System Reforms	Abbreviation	Year introduced
1	Paper based		1990’ to date
2	Pilot DHS1	DHS1	1994/97
3	National level	JEEVA	2005
4	Human Resource for Health Information System	HRHIS	2008
5	Training Institution Information System	TIIS	2008
6	mHealth Services	mHealth	2013
7	DHIS2 rollout to all district councils in Tanzania	DHIS2	2013
8	Electronic Logistics Management Information System	eLMIS	2014
9	Health Facility Registry	HFR	2014
10	Specialized Hospital, Tertiary, Zonal	MediPro, eMedical	2016
11	TeleHealth (Telemedicine)	Telemedicine	2016
12	Facility Financing Accounting and Reporting System	FFARS	2017
13	Planning, budgeting and reporting system (web based)	PlanRep	2017/18
14	Regional Referral Hospital – patient level data system	AfyaCare	2018
15	Government of Tanzania Hospital Operations Management Information System for Primary Health Care (District Hospitals, Health Centre & Dispensaries) -patient level data system	GoTHOMIS	2019
16	Afya Supportive Supervision	AfyaSS	2021
17	Health Information Mediator	HIM	2019
18	Muungano GateWay		2019

### Conceptual framework

**Fig. 1:**
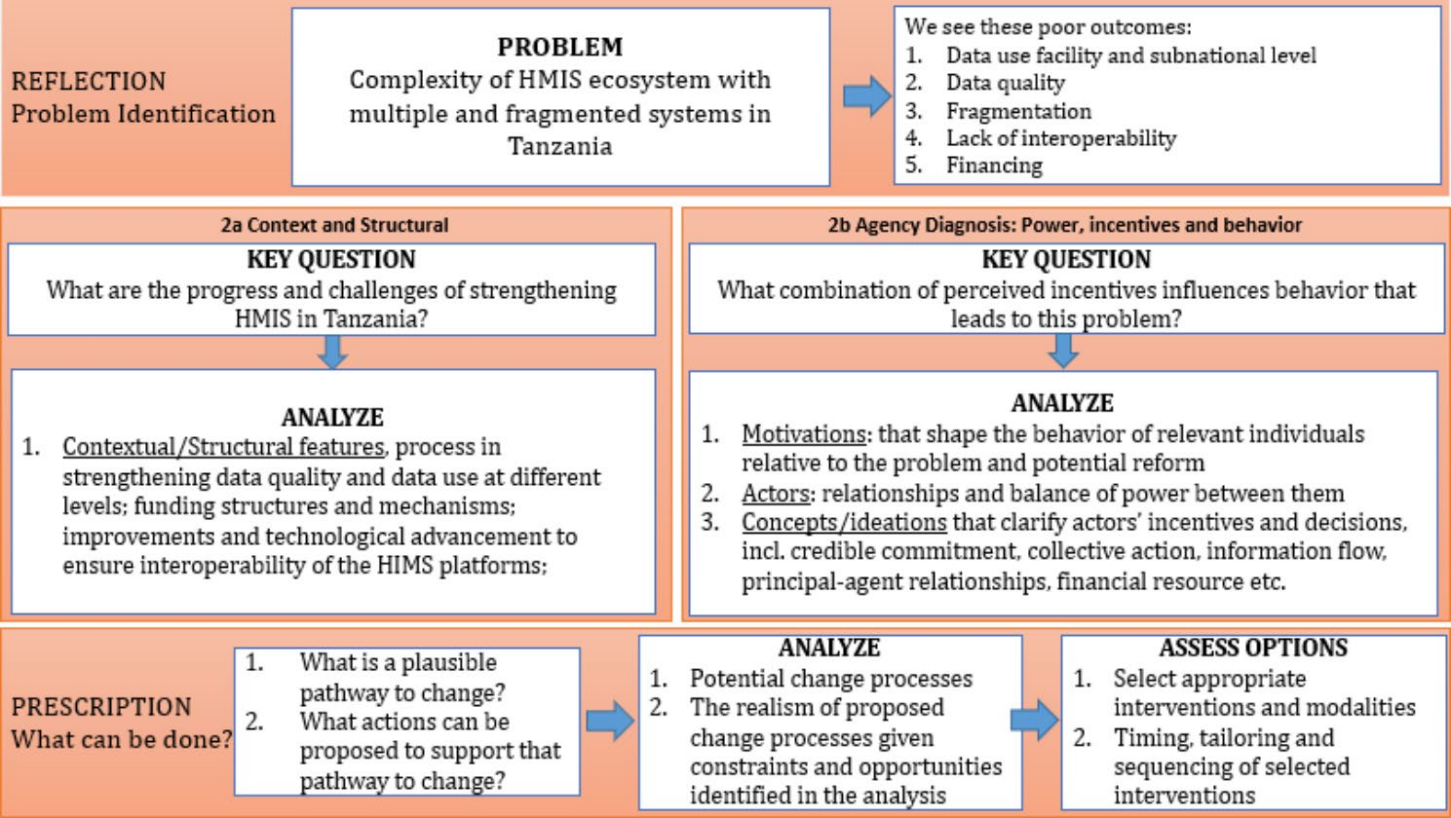
PEA Theoretical framework

The Political Economy Analysis (PEA) framework guided data collection and analysis (Fig. 1). The framework focuses on a specific problem or policy as opposed to a whole country or sector to better understand a challenging issue, the institutional dynamics contributing to the problem, the broader stakeholders, and systems factors that facilitate or hinder change [18–20]. The problem-oriented PEA includes key features such as an operational, practice-oriented nature that lends itself more readily to generating practicable, politically realistic recommendations that consider the risks of acting. Figure 1 below illustrates the analytical pathway for problem-driven PEA, as adapted from Siddiqi, Masud [21]. Both the qualitative tool and analysis were organized to align with key elements/attributes of the analytical framework: structural diagnosis, agency diagnosis, and pathways for change.

### Study design

We used qualitative research methods which incorporated data collection through document review and key informant interviews (KIIs). In particular, key documents from government, development partners, and non-government organizations were reviewed to understand the current HMIS ecosystems in terms of progress and challenges. On the other hand, qualitative interviews with key stakeholders were conducted to supplement the document review by identifying who and what affects the implementation of HMIS in Tanzania.

### Study participants

We purposively interviewed several stakeholders (Table 2) such as government officials in either health system strengthening or HMIS. These included representatives from the Ministry of Health (MOH), Presidents’ office -Regional Administration and Local Government (PORALG), and the Ministry of Finance and Planning (MOFP). Other study participants were representatives from development partners like the Deutsche Gesellschaft für Internationale Zusammenarbeit (GIZ) Tanzania and the United States Agency for International Development (USAID) because they have been supporting the HMIS ecosystem in Tanzania. Representatives from technical agencies were also involved, including the World Bank and World Health Organization (WHO). Technical agencies also included academia such University of Dar es Salaam (UDSM), Muhimbili University of Health and Allied Sciences (MUHAS) and the National Institute for Medical Research (NIMR). Additionally, HMIS implementing partners were involved (Table 2), including PATH (formerly known as the Program for Appropriate Technology in Health), Public Sector Systems Strengthening Plus (PS3+), Global Health Supply Chain (GHSC), Tanzania Mentors Association (TMA), Association of Private Health Facilities in Tanzania (APHFTA) and Benjamin Mkapa Foundation (BMF).

**Table 2:** List of Respondents by Institutions and Agencies

S/N	Stakeholder category	Stakeholders’ institution/ agency	Sample (N = 17)
1.	Government stakeholders -Ministries	• Ministry of Health (MOH) • Presidents’ office –Regional Administration and Local Government (PORALG) • Ministry of Finance and Planning (MOFP).	5
2.	Development partners (DPs) and funders	• GIZ -Tanzania • USAID • WB	3
3.	Private Institutions and NGOs	• APHFTA • TMA	2
4.	Implementing partners (IPs) and Technical Agencies	• PATH • GHSC • PS3+ • BMF	4
5	Accademia and Research institution	• NIMR • MUHAS • UDSM	3

### Data Collection

*Desk review*: The study team gathered and conducted a scoping review of various documents to understand the HMIS ecosystems in Tanzania. These documents were collected from multiple sources including government authorities, ministerial level, development partners/donor, non-government organization, and from online databases for grey and published articles or reports. Some of the documents reviewed included: Data Dissemination and Use (DDU) strategy (2015–2020) [22] and assessment report; National eHealth Strategy (2013–2018) [23]; National Digital Health Strategy (2019–2024) [24]; Guidelines and Standards for Integrated Health Facility Electronic Management Systems [25]; Health Sector Strategic Plan (HSSP) V [26]; Monitoring and Evaluation (M&E) Strengthening Initiative (MESI) [22]; and National Data Quality Guideline (2016) [27].

*Qualitative interview*: *A s*emi-structured interview guide was developed to capture information through key informant interviews (KIIs) (please see annex 1: Data Collection Guide). Qualitative data through KIIs intended to capture a variety of aspects/ themes around who and what affects the implementation of HMIS, and strategies to overcome the challenges facing the HMIS ecosystem in Tanzania. The KIIs were led by two experienced researchers: interviewee and a note taker. KIIs were conducted in Swahili, with few KIIs in English depending on respondents’ preference, while tape recording all the interviews. The tape-recorded files were saved in an online server on a daily basis to minimise the risk of data loss. Summary notes were developed on a daily basis.

### Data management and analysis

The analysis started by critically reviewing and extracting key information about various themes related to description of the HMIS ecosystem in Tanzania. This captured what has changed/ not changed around HMIS ecosystem over time, who and what influenced those changes/ absence of changes. The extracted information was eventually synthesised thematically. Prior to qualitative data analysis, all the audio recorded interviews were transcribed into text and imported into NVivo software for coding and processing. Qualitative data from KIIs were analysed using the thematic content analysis, whereby individual responses were identified and categorized into common themes. All transcripts were analysed in Swahili language, but key quotes to be incorporated in the report were translated into English language.

## Results

The findings are presented in line with the architecture of HMIS in the country. As indicated earlier- HMIS involves different players and stakeholders and thus, it was critical to analyse their perspectives and interests that drive their investment and influence on HMIS in the country. The findings are reported under the following themes; actor role in HMIS, relationship among HMIS actors, Main motives/interests among actors in HMIS, and ideations.

### Actor role in HMIS

#### Government

The analysis showed that the government is the main actor within the HMIS ecosystem in the country and is responsible for identifying the need for improvement of the HMIS ecosystem. The custodian of the main HMIS is the government. The findings showed that the role of the government has been to identify the need for improvement of the HMIS ecosystem and this has been vested within the Monitoring and Evaluation department (M&E). During the identification of the needs, the government works closely with different stakeholders to comprehensively capture the actual information needs and automate to facilitate data capturing, analysis, and access as pointed out by one of the key informants:

*“…. our role as a government is to come up with the need to automate things……. and this is mainly done by the M&E department, they are the ones to come up with the need, tool, and all other approaches., This is our role on the side of the government …… we work closely with stakeholders to ensure and help to automate their business processes….”* KII − 17, Government Representative.

Most of the key informants acknowledged the role of the government as the main stakeholder in providing strategic direction and guidance for the HMIS ecosystem. During the interview, stakeholders from the private sector pointed out that the government works closely with other stakeholders including development partners (DPs), implementing partners, private institutions, NGOs, academic/research institutions, and others. In view of the key informants’ interviews from the Private sector and NGOs, the role of stakeholders has always been to complement the government efforts as asserted by one of the key informants:

*“…. all stakeholders work to complement government efforts, so if we want to have a sustainable result we must contribute to what has been initiated or what has been planned in the Government’s strategic plan or priority, so if you look at one of the strategic objectives of the health policy is to strengthen the health management information system, then be assured that every partner should in one point or another should ensure that even their community activities, community data, every structure that they have put in the community all complements to the government strategic objective….*” KII − 6, Private and NGOs.

#### Development Partners (DPs)

Development partners were reported to provide financial resources based on Government priorities. It was noted that discussion is made with the government representatives and agreement is made on different areas of support. It was further highlighted that the DPs who are supporting the government via Basket Fund, do not make direct investments in the HMIS ecosystem. DPs usually do not direct the government to invest in certain areas, rather they do provide financial resources. This was noted in the discussion with the DPs:

*“…… we discuss and agree with the government about their priorities, then we give funds through the Basket, and then it is used to support HMIS activities…we do not tell the government to do this and this, no, we just send the money to the Basket Fund.”* KII − 12, Development Partner.

#### Implementing partners (IPs)

During the discussion with HMIS implementing partners, they reported providing technical support to the government on matters pertaining to the HMIS ecosystem. In the process of providing technical support, they usually review the existing government priorities such as HMIS investment roadmap/strategy as a guide, as commented by IPs the representative:

*“…our role is to provide technical support to the government, we play a big role in providing technical assistance in development interventions, for example look at the roadmap, let’s say enterprise architecture, we act as advisors, we provide advice on how everything will come together as a system…* KII − 14, Implementing Partner.

#### Academic and research institutions

Academic and Research Institutions were mentioned among the HMIS ecosystem stakeholders. During the discussion, it was reported that they have also been providing some technical assistance at the councils and healthcare facility level. Furthermore, it was pointed out that they have been assisting in providing training for those who are using the HMIS platforms.

*“…we actually provide support but we are more in development. For example, in case there are malfunctions within the HMIS ecosystem, we are consulted to go and resolve the problem. In addition, there could be a need to update the system, to add/reduce a certain feature in the system, then we support. Not only that but also providing training to those who are going to use the system or there are new staff who have come in, so they need to be trained, so we are going to do training for them to get to know the system….* KII − 9, Academic and Research Institutions.

### Relationship among HMIS actors

#### Relationship between Development and Implementing Partners

The majority of those interviewed pointed out that there is a good relationship between Development Partners, donors, and Implementing Partners. In the discussion with the implementing partners, it was noted that they usually work together with the donors and DPs. The intention is to ensure most of the things are coordinated and harmonized in a clear manner. When discussing the relationship between donors and implementing partners, this was said:

*“…we also work closely with XXX as a technical digital partner, therefore, our big role is to ensure that most of the XXX investments are harmonized …* KII − 14, Implementing Partner.

#### Relationship with the government

In all the interviews, it was reported there is a good relationship between the government, Development Partners, Implementing partners and other stakeholders. It was further revealed that at times DPs and IPs have formed the teams which constitute members from the government ministries to facilitate the implementation of the HMIS activities. In discussing the relationship with the government this was highlighted:

*“……. our relationship has become very good. We are working very close with the government, …… so at some point we came to realize there was duplication of efforts or there was a lag time in getting answers, so we agreed to form some sort of a team so that together we can complete our work and whenever we go to discuss with the government, we sit as one team, because we have a solution of how to support this, so that we can do the work ……. we work together here and there and the government is there…* KII − 16, Implementing Partner.

Likewise, a similar note of the existence of a good relationship between the government and other stakeholders was noted in all the KIIs with the government representatives. It was evidenced that the government has been working closely with the different stakeholders in investing in the HMIS ecosystem. In discussing how the government has been working closely with the other key stakeholders this was pointed out:

*“……. the government does sit down with stakeholders when developing health Sector Strategic Plans, and among the areas which were highlighted is strengthening of availability of data and that’s why the government saw the importance of establishing an M & E department which was given a responsibility of ensuring data is available.…* KII − 1, Government representative.

### Main Motives/interests among actors in HMIS

#### Data needs

Almost all the stakeholders interviewed pointed out the “need for the data” to be the driving force toward investment in the HMIS ecosystem in the country. Data needs emanated from the Government, the DPs, IPs, and others. It was observed that the challenges arise when interested partners feel that they do not have freedom to operate and manipulate the system. Hence, they may come with a different package and inquire about developing another system for data capturing to fulfil their needs. In addition, those investing in the HMIS have been thought to have lobbying skills to the extent that they are able to penetrate their ideas and invest where they have interest. Data needs were linked to the nature of the HMIS ecosystem in the country, whereas most vertical programs were reported to operate outside the HMIS ecosystem. They further highlighted that these institutions which need data, usually provide some technical or financial support to ensure they get access to the data. When discussing data needs, this was said:

*“…. it is the government because she wants to have information, the right information to guide decisions. For example, we must have the data when we are planning for the budget, and when we are meeting with stakeholders and development partners, also when we want to do advocacy, we must have data …… so even the stakeholders supporting us they have their areas of interest such as TB, some support HIV, and some RCH, so it depends with the interest of the stakeholder……”* KII − 1, Government Representative.

*“…… to have access to data, …no one, not even Bill Gates himself will bring you million dollars, at the end of the day that money will not be for free, he knows exactly he is going to get data for his interest. So, the motivation there is access to data of course, while providing resources to help you to collect the required data…. it is a win-win situation.…….”* KII − 10, Academia and Research Institutions.

In a follow-up discussion with the HMIS implementing partners, the same notion was made in terms of the failure of the existing HMIS to provide whatever data is needed by the partners and hence the need to start a new system. Those investing in HMIS are pushing not only because of the money they have invested but also because of the results that HMIS yields. It was further highlighted that if one cannot change the government view then at times, they tend to create a parallel system to meet their needs. Therefore, the inability for established systems to respond to the needs of partners was pointed out as a major challenge which led to multiple concurrent systems. In discussing the inability of the existing system to meet the needs, this was said:

*“…. you know that partnership is not easy. Therefore, most of the time if the system cannot provide what I need then I start my own, if the system is not responding well then, any individual can establish their own system and the rest will follow along…….”* KII − 14, Implementing Partners.

#### Technological advancement

A few of the stakeholders interviewed pointed out technological advancement as a motive for initiating HMIS. Participants from the academic and research institutions pointed out that technology has been changing over time and has impacted the HMIS ecosystem in the country. They noted that some of the systems were installed a while ago and now cannot meet the current needs of the country. During the discussion, it was noted that at times technical experts do visit the country and provide advice on innovations within the HMIS platforms and get some time to highlight the technological advancement and the need to update the HMIS ecosystem. This was detailed in the discussions with the academician and research institution as follows:

*“… there are some systems, we have here that are outdated; therefore, there are people who usually come here to provide technical advice on how we can update the systems, how to move from that stage, so that we can place them to another place which aligns with the current world. For example, you find many of the systems used here being open-source software, and you find that those open sources were installed six or seven years ago, so normally it causes problems…….”* KII – 9, Academia and Research Institutions.

#### Pilot schemes

A few of the stakeholders interviewed reported that the existence of multiple health information systems has been influenced by the existing pilot schemes in different areas. It was reported that some HMIS stakeholders have technical know-how which they want to pilot in the country. Some are extremely specific, for example, a donor wants to support immunization in the country, and there is no information/database with information on the vaccination within the country. They see the opportunity to invest in the immunization database as they have funds to invest in those systems.

“*……. every system has a specific type of data to collect for example one particular system can collect data from the health centre, another system can collect data from a regional hospital and another system collects data from a national hospital, there could be another system that collects data in private hospitals, so all in all there are several systems ……we had an agreement that stakeholders and implementing partners in our country are allowed to come in with their own systems to support the government because as the government we were trying to see what kind of system will be suitable for us in implementing data issues at an affordable costs and reliable…”* KII − 1, Government Representative.

### Power among the HMIS Stakeholders

The majority of those interviewed mentioned that the government has the power to approve or reject systems which are not in line with the government priority as stipulated in the HMIS investment roadmap/strategy. The power of the government to accept or reject any changes within the HMIS ecosystem in the country was articulated during the discussion with the implementing partners. Good leadership is when one identifies the challenges and sets some strategies for improvement. They confirmed that the government is very powerful, with the mandate to prohibit anyone from collecting data, using the data as well, and using existing healthcare workers at the facilities to capture the data. When this question was asked to the implementing partner, he reported that:

*“…… the government is very powerful, and has the mandate to prohibit anyone from collecting, and using the data or even using healthcare workers in the health facilities to capture the data. It has the power to give directives on which systems to strengthen. …….”* KII − 14, Implementing Partners.

On a different note, during the discussion with respondents from academia and research institutions, it was reported that the power to accept a system is left to the hospital board. It was noted that in-country ICT staff do meet with foreign ICT staff who have various ICT systems/products as they present their system/product, someone feels that the system can help to address data needs. However, approval of the installation and operation of the system, for example in public hospital settings is vested to the hospital board which takes time to review and later on consult the government for further inspection and approval. The board usually takes time to review and assess the system before making any investment.

*“……. for example, we ICT staff usually meet with people from abroad, and they have many systems, and when you take time to check the system, you just realize that this can be a big help here in our country, but you cannot approve until the hospital board comes, check the system, and agree. Furthermore, we cannot fix that system and start using it at the workplace until the government inspects it and proves whether it aligns with government systems because they collect people’s data and should be connected and integrated directly with government databases……”* KII − 9, Academia and Research Institutions.

#### Power arising from long term relations with government

In the discussion with the implementing partners, it was reported that some of the partners who have been supporting the government over a long period of time are powerful in influencing HMIS investment decision-making compared to those who are recently coming to support. Furthermore, the power was linked to the established relationship with the government, the longer the relationship, the greater the likelihood that the partner has influence over the investment within the HMIS ecosystem. This was affirmed during the discussion with the implementing partner:

“*… there are partners who have stayed for a long time and supported the HMIS ecosystem, so they are very trusted and have worked in the government for a long time, and there are partners who pioneered projects which are currently introduced, and that their interests are big/high; however, their ability to exercise power relies on how long have stayed and interacted with government, but also their power depends on what kind people have been employed, you know there are the so-called one relations….”* KII − 15, Implementing Partner.

#### Power over financial resources

Most of the stakeholders interviewed reported that power over financial resources influenced decision-making in HMIS in most cases. Whereas stakeholders with financial resources were reported to have the likelihood of influencing investment within the HMIS ecosystems. A Development partner with more resources and the intention to implement the intervention on a greater scale would be more welcomed as compared to a partner with fewer resources. In discussing the power over financial resources and its effect on the HMIS investment decision, this is what was said:

*“…., but also, there is the issue of money, if DPs are doing the same thing while one has more money; the one with more money may have more influence; let’s say one DP has funds for implementing in one region while the other DP has fund to implement maybe in twenty regions; obvious the one with more money will be given a priority, has influence….”* KII − 15, Implementing Partner.

#### Coordination of financial resources

In most cases, the coordination of the financial resources was viewed to be vested within the government financial controls. Whereas it was reported that most of the resources are channelled through the Basket Fund, where funding resources from different donors are pooled together and allocated for various government plans including the HMIS ecosystem. However, there are some donors who channel some financial resources directly into the system in close consultation with the government.

*“…. there are financial resources that go straight to the government, for example, most of the money is pooled together within the Basket Fund whereas some are for the improvement of health management information system; therefore, it means that is managed by the government, but there are others the donor who wants to manage the funds themselves for some reasons….*” KII − 7, Private and NGOs.

In a follow-up discussion with the development partner, it was made clear that the government plays the greater role in coordinating the resource and it is accountable to the development partners. There are two channels for financial support, one is through the implementing partner while the other is direct channelling the money to the government and include the commitments in the Medium-Term Expenditure Framework. It was noted that the donor comes via partner means the implementing partner, so the money is kept by the implementing partner. In such case of direct financial provision, the government must provide a financial report to account for the funds received. The financial resources are managed using government financial management systems such as EPICOR and “*Mfumo wa Ulipaji Serikalini* (MUSE)” as a new Government expenditure system and one can trace the funds in the system. In explaining the coordination and accounting on the financial resources directed to the government this was said:

*“…. there are different types of management, the one is when a donor provides funds directly to the government, like XXX provides funds to the Ministry of Health, so it means the Ministry of Health will implement and submit financial report to that donor; like that….*” KII − 15, Development Partner.

#### Power during technical working groups

Most of the stakeholders interviewed noted that the government has initiated the technical working groups where stakeholders (government, private, technical and donors) have the forum to discuss and harmonize the ideas. Participating institutions in the meetings have equal opportunity to discuss and give their opinions. Furthermore, during the meetings there are strategic plans which are being discussed including the HMIS and if there are changes which the government is planning then they are discussed. Through these forums people get to know what is being done by the government and other partners. If the systems were working properly there would not be any overlap. It may happen that development partners and others providing technical support if they are not participating in such forums and fail to know government priorities as a result, they end up thinking in silos.

*“……all the stakeholder has an equal opportunity of providing their opinions of what should be done, usually there are technical groups, there are Sector-Wide Approach (SWAP) meetings….”* KII − 12, Development Partner.

It was noted that the ministries responsible for the HMIS ecosystem have equal opportunity during the meetings. The two ministries MOH and PO-RALG would be leading the meetings, where one becomes a chair and the other a co-chair. They are the ones coordinating the technical working group meetings.

*“… when we have meetings, we all meet as health stakeholders and of course, every department has their own partners and stakeholders, for example, partners in family planning, have their own group, partners in HIV have their own group, partners in TB have their own group, …. we coordinate all these implementing partners in case of any amendments in the data area, after that, we will have another SWAP meeting and present every other concern, of which from this meeting there will be a presentation and report of an agreement for a better system to be used. On the side of ICT too there are technical group working which also has their meetings on what system should be used however they are guided by IGA, it’s the one that guide and aid in a better system that can help the country, and on our side of Statistics we are guided by National Bureau of Statistics, it’s the ones that approve which data to be used, at times they tell us on what data should be included and another one excluded…”* KII – 1, Government Representative.

### Concepts/ ideations

#### Integration and interoperability

The majority of those interviewed pointed out that the whole process of integration and interoperability has taken more time than was envisioned. Among the issues raised during the discussion were linked to the fact that the systems did not grow together. Initially, these systems were allowed to be piloted and scaled up. The challenge came at the time of integrating and ensuring interoperability as at the very beginning implementers had not agreed that they would later integrate them into existing systems. During implementation, each one had her own ways of implementing and operating the system. In the process of integrating the systems, each one realizes that there are some things that one must do so that the systems become operable.

*“…… these are projects which were designed and implemented/piloted separately and therefore bringing them together may take some time. It will take more time as the landscape for the systems was quite different……”* KII -, Implementing Partners.

#### Open-source vs. closed software

In the discussion with the academia and research institutions, it was pointed out that among the challenges to the integration and interoperability of the HMIS ecosystem is the nature of the software’s. There are open and closed software’s. Whenever one wants to make changes in these software’s to fit in their context it becomes a problem and at times, they have limited financial resources. It was pointed out that the closed software providers sometimes demand an enormous amount of money to assist in fixing problems. It was noted that open-source software is easy to access and ensures integration as compared to closed ones.

*“… I think the main thing causing slow integration is the nature of the software, the software provided to us as assistance was not open source but closed software; therefore, you cannot access and fix respective an inter-processor interrupt (IPI), it is upon on themselves to take the responsibility to fix or provide certain code so that one can fix that IPI. Sometimes they demand money, which means they want an excessively big amount of money to fix the system ….”* KII − 9, Academic and Research Institutions.

#### Technical capacity and advancement

Participants from the ministries claimed that they have enough technical capacity now to support some of HMIS platforms in the country as they have experience with the existing systems. It was reported that existing staff have skills to support the systems. In the discussion, it was reported that the plan for integration and interoperability when implemented will not lead to closure or data loss for any institution, rather the intention is to streamline the data collection tools. Furthermore, the purpose is to reduce the workload to the healthcare providers who are responsible for the data capturing and entry into the system (tedious work performed by the healthcare providers).

*“…. I must admit that technology is still an issue because you know technology is unlimited, technology itself is insufficient even Bill Gates and his team are in the lab innovating a system which will facilitate his market, so we are trying not to implement but we are still not competitive enough in technology ….”* KII – 1, Government representative.

In a follow-up discussion with one of the DPs, it was pointed out that the government has staff across Tanzania with technical capacity including computer programmers and network engineers. The government can make some progress using its internal capacity. When discussing, this was highlighted:

*“……in my experience you may find that the government has its modules, it shows the motive that we would like to do this within this timeframe. Since the government has shown some directions, as stakeholders our job is to help the government implement that intent according to the government’s needs…. what is needed is intention and the available capacity. For example, in the area of systems you will find that all the systems that we are working with have been established by the government, the government has computer programmers, network engineers, in all district hospitals there are IT technicians who can do those works.….*” KII − 13, Development Partner.

Contrary to the views of the other stakeholders, academic and research participants noted that the lack of human resources with the technical know-how to operate the system is the driving force towards slow integration. Respondents noted that it is important to ensure those working as well as leading the ICT departments have skills and knowledge on the ICT ecosystem. This will make the integration and interoperability of the HMIS ecosystem run smoothly. This was highlighted during the discussion as follows:

*“…. As I said, politics is everywhere even in the health sector, same as politics in the ICT section. You may find in the ICT department there is no person who is fully trained on the existing systems. You may find someone who does not understand the ICT ecosystem very well [….is heading the department…]. But if someone with a deep understanding of how the whole system of the institution functions, is assigned to the ICT department will match with it and will be able to make things run smoothly without any problem….*” KII − 9, Academic and Research Institutions.

#### Stakeholders resistance

In a few of the interviews conducted it was noted that at times there was some resistance from stakeholders to work together within the HMIS ecosystem. Initially, at the healthcare facility, one would find about three computers at the records office, one for the Care and Treatment (CTC) program for HIV-positive patients, another one for the malaria program, and another one for United Nations International Children’s Fund (UNICEF). Whenever the records officer wanted to perform some CTC work, she had to switch on the CTC computer, and could not use a different computer for another program. One computer could be used to perform all the tasks. Participants were of the view that there could be some resistance from different stakeholders supporting these systems. In addition, stakeholders supporting these programs sometimes have set the budget for their activities. There is no flexibility in such budgets whenever there is a need for changes, they are bound by their contracts.

*“…. if the donor had already given you funds and supported you with technical expertise and you innovated a system and trained people and continued to use it. If you want to remove that system ………* [without discussing with the funder] …. *the funds start to complain, so I think the donor will be difficult to accept. For example; a donor who supports that programme in multi-countries then that is the same system being used to collect data, so if you want to remove that system of course she will be hesitant especially since she uses it every other place to collect data through that system….….*” KII − 8, Academic and Research Institutions.

*“……As I said earlier the systems are developed with the help of partners, so when you want to integrate if partner A is not interested with regard to the system that she supported but she is not ready you will therefore get stuck.… when you want to join systems, you should remember that those systems were developed by different partners, and of course the health sector is a stakeholder but we also have specific stakeholders of whom you will obviously need them to meet with them for all to give their ideas………*” KII − 10, Academic and Research Institutions.

#### Shortage of financial resources

In the discussion with the private and non-governmental organizations, it was evidenced that the shortage of financial resources has hindered the whole exercise of ensuring integration and interoperability of the systems. It was reported that most of these systems have been financed and technically supported by donors for some specific reasons. There are a lot of processes requiring enormous resources to ensure integration and interoperability of the existing HMIS ecosystem.

*“…. there are many projects which might have been connected and which have been donor given …. to ensure integration, there are a lot of activities which need funds, there is the issue of orienting people, there is the issue of inviting experts for technical support, like that and then is to conduct dissemination of what you have done, therefore all those need funds, ….*” KII − 7, Private and NGOs.

“….*I think the big issue here could be about the finances because when you want to integrate systems, you should remember that those systems were developed by different partners, and of course the health sector is a stakeholder but we also have specific stakeholders of whom you will obviously need them to meet with them for all to give their ideas; if they see it as a simple thing and can be done but it is quite expensive. So, I think finance is the major reason because it is very open and the rest are politics influenced by finances.….”* KII − 10, Academic and Research Institutions.

#### Lack of political Will

In one of the discussions with the participants from the private and non-governmental organizations it was noted that the lack of political will has been a hindrance towards integration and interoperability of the HMIS ecosystem in the country. This was attested during the interview with the representatives from the Private and NGOs.

*“…. there is limited political will …… so maybe the issue is for the government to continue being aggressive, and maybe the government itself to take a lead, and for to them keep money aside for facilitation of interoperability and not to wait for projects…. Government should come to the frontline to support and maybe they have to put a timeline, let say when it reaches this date, the integration thing must get finished….*” KII − 7, Private and NGOs.

In a different discussion with the implementing partner, it was reported that there are lots of activities going on and sometimes prioritization of the activities is lacking behind. Every day staff working within the government are driven by today’s needs and not overseeing the overall need for the future. In that situation it was noted that it will take some time for integration and interoperability to be realized.

*“……for those that have worked in the government they understand when I say to priorities, for example of priorities is that, I am going to the office with six things on my mind, when you reach at the office you are called by the Minister and then she gives you directives that are of his focus, by tomorrow you already have 10–15 things, and people are so busy to respond to current issues, so the idea of thinking and going ahead is being crumpled because they are too busy ………” KII − 15*, Implementing Partners.

Contrary to the notion from the other stakeholders about the slowness of the integration and interoperability of the HMIS ecosystem, government representatives believed the existence of multiple systems is because at times people are afraid of having only one system throughout the country. This is what was reported in a discussion with the government representative:

. *never know the capacity when we are doing interoperability, at times we ask ourselves if the system is effective to serve the whole country or is there any time when the system will collapse and have to start everything fresh, so that is our consideration, that is why we are taking everything step by step.” KII − 1, Government Representative.*

#### Lack of power to question

Respondents felt that some of the staff and departments in the ministries have no power to question the operations of the HMIS ecosystem. It was reported that staff working for HMIS do fear to argue as they might be reported to the higher authority. On the other hand, it was noted that donors have the power to question HMIS coordinators and managers, while the HMIS managers can hardly question the government.

*“……at times donors can challenge the HMIS manager but the HMIS manager cannot challenge or argue with the Minister, so there is an inability to focus which originates from the system. As a result, you become reactive every single day and you cannot think about the future ………” KII − 15, Implementing Partners.*

#### Lack of coordination

In the discussion with the implementing partners, it was noted that within the government at times different departments do compete for resources to the extent that there is no coordination of the ideas as well as available resources. The lack of clear coordination within the ministry and inter-ministerial extends the problem to even coordinating the financial resources coming from the Development Partners. This was highlighted in the discussion:

*“…… I think as I said, the first thing is collaboration within government, every unit wants to get funding or say a certain department, they think getting funding is a process; they do not have a collective decision that we have to sit and agree about this system. Therefore, everyone follows his way; but also, many donors like to, each donor would like recognition let say I have done this and this; but even for donors, the coordination is a challenge, means that are coordinated! This donor brings this money, this person passes this way and this through that way, you see therefore even donors themselves are not well coordinated, to know which should bring in, you see.………” KII − 15, Implementing Partners.*

The discussion went further highlighting the existing challenge in terms of involvement of various stakeholders in the meetings where they have an opportunity to discuss the matters pertaining to the HMIS ecosystem. It was clear that few stakeholders could engage in meetings and interaction with the government. Involvement of the development partners, implementing partners, government and its units, and others, would have given an opportunity to understand whatever is going on in the country and stakeholders could leverage their resources instead of initiating new ideas.

#### Coding and labelling of the Systems

In one of the interviews, it was highlighted that there are two major ways to ensure interoperability of the systems. One of them is peer-to-peer; where system A links to system B straight like connecting one computer wire to the other computer. The other approach is connecting the systems using a junction box so that if there is a third computer it can connect via the junction box. For example, “*Muungano”* Gateway has been linking the different systems. The coding and labelling of variables within the systems has also been a challenge in ensuring interoperability. For example, one system recognizes sex as male and female, while another system recognizes sex as F and M, and the third system sex is coded a 1 (male) and 0 (female) but they refer to one attribute. When sharing such information, the values must be translated in such a way that data coming from any of these systems will have the same meaning as the other system.

#### Maturity of the existing Systems

In one of the discussions with the development partners, it was reported that some of the existing systems have not been developed to their full capacity. This has made integration and operability of the system to be slow. The failure to operationalize the systems to capture required information at the health facilities was linked to the shortage of resources. In discussing the maturity of the system and the shortage of resources, this was explained:

*“…there are factors that are within, and there are factors that are beyond; all these takes time, takes money and a lot of them they are out of the control of the health sector; so you just have to wait by sometimes do a small scale until you can coop,… integrate some few systems, wait for other systems to have maturity; others have little maturity so need some time, over and back to you.….*” KII − 11, Development Partner.

A few stakeholders were concerned with having one system for the entire country. However, it was viewed from the government perspective that having a single system for the entire country is very risky in case of malfunctions in the system. One of the government representative institutions believed they do allow multiple systems for the purpose of ensuring whenever one system collapses, they do have the backup that is why dispensaries and healthcare centres have *GoTHOMIS* in place, while regional hospitals have been implementing ‘*AfyaCare’* as systems for capturing the patient level data.

*“….so sometimes as an expert you can argue yourself to see what is best, is it better to have one system that will oversee all of this or to have specific systems for a specific aspect, what will happen if the system crush and cannot function, …… sometimes we look after the security and safety of the country, we have not had a vivid example of a country that has managed to have one system in the whole country….….*” KII − 1, Government Representative.

#### Network connection and infrastructure

A problem with network connection was noticed to slow down the integration and interoperability of the HMIS ecosystem in the country. During the discussion with the government representative, it was clear that there are some areas in the country where the network connection has affected the HMIS ecosystem as a result healthcare worker end up using paper rather than digital systems. This is what was reported during the discussion:

*“…. in using the system but there’s no network, in some remote areas there’s a network problem and the system delays in responding so one just decides to abandon the system and use manual way of filling information, so those are the issues on ground and we are working on them to ensure we create systems that are parable to everyone….”* KII – 1, Government Representative.

In a few of the interviews it was reported that infrastructure has been a major challenge within the HMIS ecosystem in the country both the equipment, hardware, software as well as internet connectivity in remote areas. It was noted that at times staff from the healthcare facilities must submit such information manually.

*“……… infrastructure yes, there is a challenge specifically in rural areas, because of the issue of connectivity. One is supposed to submit this information manually, maybe you are required to send them via email, and there is no internet in other areas, so that’s one and also even the line, just to say about village areas; you visit a health facility you may wonder, some staff uses their own infrastructures, but let me say that generally, the presence of that infrastructure without considering whether is from government or what has been helpful, but the big effort needs to go to the low level, it is where many information come from, in dispensaries, health centres, for district hospitals, not bad, not very bad but when you visit health centres and these dispensaries, frankly still there is a very big issue of infrastructure…”* KII – 7, Private and NGOs.

### Recommendations

Table 3 summarizes the major challenges and provides some recommendations to improve the HMIS ecosystem in the country from the participants’ perspectives. The challenges emanate from interrelationships among HMIS ecosystem stakeholders, power differences, technical capacity and technological advancement, lack of political will, maturity of the existing systems, and low integration and interoperability process.

**Table 3:** HMIS Ecosystem Challenges and Recommendations

Challenges	Recommendations
Power difference	1. To harmonize power difference among different actors, the government should have priority in place then all donors, technical and implementing partners will be informed during the meetings. In addition, each stakeholder interested in investing and strengthening the HMIS ecosystem should then be given an opportunity to choose among the existing government priorities.
Power in decision making	2. Decision making on initial investment and strengthening HMIS ecosystem should consider different stakeholders who has shown interest with HMIS ecosystem but the government should take a lead. More involvements of key stakeholders should be considered at the initial designing, execution, and evaluation of the interventions within the HMIS ecosystem.
Technical Capacity and Advancement	3. The government should continue welcoming development and technical partners who are able to capacitate staff on various platforms to ensure integration and interoperability of the systems. As it was noted that ministries have staff who are able to operate technical but lack modern technology and equipment’s for some of the systems
Stakeholders Resistance	4. It is important to engage every stakeholder interested in the HMIS ecosystem by bringing all of them together, discussing and making some plans for future improvement of the HMIS. 5. It is important for the responsible departments at the ministries to call together all the partners in one place and brainstorm together. This will give the opportunity to identify all the data gaps existing for various interventions on going into the country and be able to work together towards integration and interoperability of the systems.
Shortage of Resources	6. The government has deployed ICT personnel in the councils, as well as ensuring availability of office space at the council levels. The government in close collaboration with development partners, technical and implementing partners should ensure the offices are equipped with modern equipment. 7. Stakeholders interested within the HMIS ecosystem in the country should continue to allocate resources (financial, equipment and technical know-how) to aid/supplement the government efforts. 8. To strengthen the HMIS ecosystem in the country there is a need to continue using local experts who know the country’s environment and context. 9. The use of local experts helps to contain costs for strengthening the HMIS ecosystem, though there is a need to build their capacity for sustainability purposes as some of the pilot schemes have been phasing out now and then.
Lack of Political Will	10. It was noted that already there is a circular with instructions from the Hon President that systems available in the health sector should speak to each other. It is recommended that all stakeholders interested in investing and strengthening the HMIS ecosystem should be coordinated to invest in the areas which have already been identified by the government.
Lack of Coordination	11. The government has in place some of the strategic documents such as HMIS investment roadmap where mechanisms for investment/strengthening HNMIS are clearly stipulated. It is important to ensure information contained in the documents are clearly communicated to each stakeholder interested in the HMIS ecosystem in the country. 12. There is a need to strengthening coordination within the ministry and inter-ministerial, ensuring there is a platform where stakeholders can meet and discuss things together
Coding and Labelling of the Systems	13. Capacity building should not only be at the national level, but also at the sub-national level to ensure staff are conversant with the systems
Maturity of the existing systems	14. There is a need to roll out the systems holistically within the healthcare facilities and across levels of care. Meaning that within the facilities all the departments which need to capture information have required tools/equipment to capture information. In addition, systems must be implemented not only at the hospital settings but also at the health Centre as well as dispensary.
Network Connection and Infrastructure	15. Government plans have already been identified and there are policies in place, therefore, the government should make health a priority and set aside funds for improvement and strengthening network connection and the entire HMIS infrastructure.
Staff motivation	16. Motivation of staff working within the HMIS ecosystem in the country is of paramount importance, even though they are being paid salaries and other allowances.
Low integration and interoperability process	17. There is a need to ensure that all initiatives, for HMIS integrations and interoperability serve the intended purpose. There should be post implementation monitoring of the resources invested in strengthening the HMIS ecosystem to ensure value for money. Also, outputs should be monitored to see the actual results of the actual implementation, this will help stakeholders to double check for accountability purposes. 18. Integration and interoperability would reduce duplication of efforts as well as uneven distribution of limited financing resources going to the health sector in the country. 19. For a successful HMIS systems interoperability, integrating peoples/departments (Political will) within sector ministries would expedite the process. 20. Integration and interoperability would allow comparison and standardization of health information generated from the healthcare facilities through the country to inform the decision-making process. 21. Ensuring full integration and interoperability of the HMIS ecosystem will help to alleviate the problem of fragmented tools and indicators which have been created by disease and funding sources in the country. This will also serve the purpose of reducing the workload at the healthcare facilities where healthcare workers have to fill similar information in different systems.

#### Capacity building

Participants believed there should be a continuous process for capacity building for the staff working within the HMIS ecosystem in the country. Capacity building should not only be at the national level, but also at the sub-national level to ensure staff are conversant with the systems. There is a need to review human resources working within the HMIS ecosystem in terms of their performance, the people should settle and look at the human resource system on how they can be able to perform for its interoperability if not the integration. Improvement in HMIS should be done in a holistic approach, in the case of interoperability of the human resource system or the immunization system the focus is not on the software, the focus should be on the practices done by those people. Capacity strengthening for the HMIS at various levels up to the facility level should be considered. It was reported that in each district there is a HMIS coordinator but there have been some doubts in their capacity to work within the HMIS ecosystem. This was narrated as follows:

*“…. I think integration is one thing, to me what is important is continuous capacity strengthening to the HMIS coordinators at the council level. Also, capacity strengthening for the HMIS focal person at every facility because this is what is missing. Despite the fact that each district has a HMIS coordinator, trust me when you take twenty councils randomly and bring them; I tell you among the twenty coordinators you may find that more than 40% of them or even 60%, their ability to pull data from the DHIS2 is a challenge….*” KII − 10, Academic and Research Institutions.

In a follow-up discussion with the implementing partners, it was evidenced that the capacity building should cut across all the level, national level, regional, district and facility level. When discussing the issue of capacity building with the implementing partner, this was highlighted:

*“….capacity building should be carried out to experts not only IT experts but business professionals as well so that they can engage in reprioritising and improving the needs, capacity building should also be done to HMIS and PSU experts, however this is no longer at a reporting rate its outdated right now we should seek for something that will activate and motivate need of the hour as we will automatically we will be able to leverage digitalisation to a better and more written data.….*” KII − 16, Implementing Partner.

“*…. Like I said before in the area of capacity and skills we need skills, we welcome people from worldwide who are able to help us into acquiring new skills and to be specific technical skills, we have people who are able to operate technical but we lack modern technology and equipment for operation but we are still having some challenges on interoperability, ‘Muungano Gateway and HIMU’, we have almost hundred is system but the challenge is how to coordinate them, there are also system in private hospitals that need to be linked with the government data for uploading, another need is on the technical support we need the technical support to facilitate harmonization of activities….*” KII – 1, Government Representative.

#### Integration and interoperability

Participants from academia and research institutions believed a stepwise approach should be taken to achieve full integration and interoperability of the HMIS ecosystem in the country. It was noted that one cannot integrate all the systems at once, it should be case by case because each system that you see behind it there are historical things.

*“……I advise first, this should be case-based, it cannot have interventions to say you want to integrate all systems or to interoperability all systems. It should be a case by case because each system that you see behind it there are historical things included, so how it was established. I think it should be case by case for a one by one system…….*” KII − 8, Academic and Research Institutions.

In a follow-up discussion with the implementing partners, they noted that any investment into the HMIS ecosystem should serve the intended purpose. Furthermore, such initiatives should be closely monitored and milestones be set for each subsequent step.

*“… the main thing is to ensure that all initiatives, aimed towards HMIS improvements and integrations should serve the intended purpose, and this purpose should have post-implementation monitoring that for example, XXX was given some money for integration then he should be able to submit the deliverables and going extra miles in implementation and the output should be monitored to see the actual results of the implementation…this will not only bring accountability in implementation but also adding value, and should ensure such improvements lasts long.….*” KII − 16, Implementing Partner.

#### Strong leadership

Stakeholders interviewed believed having strong leadership and governance structures is crucial in ensuring integration and operability of the HMIS ecosystem. It reported that there should be a proper mechanism for transition from plans to implementations. Bringing together government, donors, implementing partners and other stakeholders.

*“… the government should sit down and come up with a clear workplan and prioritization, should come with clear regulations; government, donors and private sectors have to sit and come into consensus, should have one language/focus, to be approved, to be implemented, that’s the way forward…”* KII – 5, Private and NGOs.

Strong leadership and governance were also viewed as among the driving forces toward the improvement of the HMIS ecosystem in the country. It was noted that the provision of the priorities will make the stakeholders align with the government plans to the extent that the implementations will not vary across regions in the country. In addition, a strong government in place will help to harmonize power.

“*……. the government should have priority in place then those donors will be given them when coming, they have to be told just to choose means which area they want to support and not someone to come with her project, …. therefore you provide to her with your priorities which need to be implemented, but if you continue to leave free, the project owner may tell you, I am going to implement in Mbeya, that’s a challenge and maybe that problem does not exist in Mbeya, that may highly help to harmonize the power, that power and domination of decision making when comes to health management information system…”* KII – 7, Private and NGOs.

The above argument on having strong leadership and governance was seconded by the argument from academia and research institutions. They argued that it is important to engage every stakeholder interested in the HMIS ecosystem by bringing all of them together, discussing and making some plans for future improvement of the HMIS. The integration and operability were viewed positively as it will improve service provision, improve access to health services through reliable statistics. Academic and Research Institutions believe it is important to get ideas from each individual stakeholder who has interest in every system by bringing them all together after collecting all their ideas and discussing them.

“… *stakeholder engagement is very important to engage in every individual to get their opinion … bring all stakeholders together on the table and then we discuss all together. But also, you need to come up with a reason why you want to integrate the systems, what is the problem today? Are you sure the same problem you see within the health system today, you can as well see it on the communication system today? When you conduct integration how much will the problem reduce, or will they disappear? Don’t let the stakeholders discuss; each one with their own opinion, there will be so many arguments at the end of the day, but they will also be a convergence point that ok guys at the end of the day we want to integrate the systems…”* KII − 10, Academic and Research Institutions.

## Discussion

The study aimed to conduct a political economy analysis on the HMIS ecosystems through an in-depth understanding of the current situation of the HMIS ecosystem in Tanzania. It was found that the government is the main stakeholder within the HMIS ecosystem in the country and responsible for identifying the needs for improvement of the HMIS ecosystem. Furthermore, the government receives financial and technical support from DPs, implementing partners and other stakeholders interested in the HMIS ecosystem. There is a good relationship among stakeholders currently working within the HMIS ecosystem in the country. The “need for the data” has been the driving force toward investment in the HMIS ecosystem. Our study revealed that the government has the power to approve or reject systems which are not in line with her priority as stipulated with the HMIS investment roadmap/strategy. Integration and interoperability were found to have taken longer than was envisioned and among the reasons pointed out are the fact that these systems did not grow up together, in addition to the shortage of financial resources and the lack of political will.

Integration and interoperability are equally important to ensure the HMIS collects similar information with the minimal cost. Besides, it enables different institutions including the government, DPs, implementing partners and other stakeholders interested within the HMIS ecosystem to improve communication and collaboration in the most effective way [28]. Integration and interoperability are processes which are based on the need for institutional collaboration, sharing information and mutual understanding of the needs for HMIS stakeholders. It implies that each institution involved or affected by the HMIS ecosystem, during the design, implementation, and operation of the HMIS should be willing to take part in the discussion. In addition, integration and interoperability follows principles which highlight the need for formal semantic description of the models to facilitate the process from specification to implementation. Successful integration and interoperability of the HMIS ecosystem will help to alleviate the problem of fragmented tools and indicators which has been created by disease and funding sources in the country. This will also be beneficial in terms of reducing duplication of efforts as well as uneven distribution of limited financial resources going to the health sector in the country. Furthermore, such integration and interoperability would allow comparison and standardisation of health information throughout the country [29]. Improvement within the HMIS ecosystem requires accountable leaders to oversee day to day operations of the HMIS ecosystem. In addition, it requires strong governance and enough financing resources from national to sub-national level to promote the desired outcomes [29, 30].

Staff working within the HMIS ecosystem have the role to inspire and sustain professionalism that impacts the quality of services. This implies that an effective HMIS ecosystem would require an institutional structure that has appropriate staff with technical skills and knowledge to perform their task at different levels of implementations. In Tanzania, staff are available at higher levels (national, regional and district level), however, at the facility level there is still a shortage of staff. It is equally important for the staff to work collaboratively to customize the integrated platform to meet the desired HMIS needs. There is a disparity in the distribution of staff responsible for HMIS across districts in the country [7]. Furthermore, staff skills and knowledge, shortage of training, refresher training together with the lack of incentives and tools have undermined the performance of the HMIS ecosystem in most countries [31].

It is important for development partners to work together with the implementing partners and other HMIS stakeholders to devolve a strategic approach to digital health investments. For example, in Tanzania, PATH has worked together with the Bill & Melinda Gates Foundation and invested in Data Use Partnership. They further worked together with the government and engaged 180 stakeholders (from the government, implementation partners, health teams, and facilities at all levels) to develop a set of 17 priority investment recommendations with specific activities, costs, and timelines for 2017–2023 [32].

Stakeholders had referred to the government documents which guide the investment with the HMIS ecosystem. Among the documents referenced is the National Digital Health Strategy 2019–2024 which is in line with the Tanzania Development Vision 2025 and the Health Sector Strategic Plan 2015–2020. All these potential documents have made it clear that there is a need to harmonize the existing systems and ensure provision of high-quality healthcare data. Furthermore, the strategy seeks to emphasize on the utilization of digital health for better health outcomes. The National eHealth Strategy, which was issued in 2013 guided the use of ICT in supporting health sector transformation in Tanzania. The strategy has indicated key investment areas within the HMIS ecosystem and aid stakeholders to prioritise need and make investment decisions. Stakeholders pointed out that investment in the HMIS ecosystem requires a lot of financial resources. Examples of costs assessment done in the country, showed that to implement all the recommendations of the Digital Road Map would cost about USD 74 million, whereas the Gates Foundation, for example, had given USD 15 million to fund seven of the highest priority projects [33].

Primary data should be used to inform decision making which occurs at multiple levels of health service delivery in any country. However, the originality and quality of data need to be assessed before making such a decision. The use of data does inform country-level policies, planning, or program decisions, and there is the need to raise additional resources for scale-up of programs or for future programs. Data also helps to assess whether a policy, plan, or program has produced the desired or intended impacts, efficiency, and quality of services provided. Nonetheless, good data to inform decision-making needs continuous investment within the healthcare facilities as well as capacity building and refresher training for the staff involved in data generation, analysis, and interpretation at the national and sub-nation levels as evidence suggests that the necessary capacity to analyse, interpret and use data, often decreases as one moves from national level to sub-national level (Harrison & Nutley, 2010). Existing fragmentation limits data use and at times may have negative consequences at the national and sub-national levels. At the national level, if the data generated and analysed do not reflect reality, this may undermine the country’s progress towards Universal Health Coverage. Furthermore, the use of poor data may compromise the government planning and budgeting process, misalign healthcare providers incentives, as well as mistarget service to the population because resources flowing to the sub-national level may not be in accordance with the healthcare needs of the citizens. In addition, the distribution and re-distribution of human resources and healthcare commodities are informed by the use of healthcare services, therefore, fragmentation may lead to imbalances in resource allocation. Fragmentation can also contribute to health inequalities, ignoring the needs of marginalized populations, sampling bias, and distortions for studies that use data from the HMIS ecosystem.

The results presented in this report should be considered in conjunction with some limitations. First, the study was limited only to the government, DPs, IPs, academia, and research institutions. End users at facility and sub-national levels were not consulted to participate in the study. Future studies within the HMIS ecosystem should consider getting insights from the end users of the HMIS ecosystem. Exploring the quality of the data generated from the various HMIS systems was also beyond the study objective. Future studies could consider extraction of the data from the system and assess their validity and reliability in informing policy making processes.

## Conclusion

HMIS requires continuous capacity building for staff not only at the national level but also at the sub-national level to ensure they are conversant with the systems. To manage power differences among different actors, the government should have priority over all donors, technical and implementing partners will be informed during the meetings. The government should continue welcoming development and technical partners who are able to capacitate staff on various platforms to ensure integration and interoperability of the systems. Government plans have already been identified and there are policies in place, therefore, the government should make health a priority and set aside funds for improvement and strengthening network connection and the entire HMIS infrastructure. Furthermore, it is important to conduct a need-based assessment of the staff at the sub-national level to identify available capabilities and the knowledge gap to be assisted. This will improve the HMIS ecosystem in the country.

### Electronic supplementary material

Below is the link to the electronic supplementary material.

Supplementary Material 1

## Data Availability

The datasets used and/or analysed during the current study is owned by UNICEF Tanzania and are available on reasonable request.
