# Supplementary material for: A political economy analysis of strengthening health information system in Tanzania
Source: BMC Med Inform Decis Mak. 2023 Oct 30;23:245. doi: 10.1186/s12911-023-02319-9 (PMC10617168; doi:10.1186/s12911-023-02319-9)
Supplement: Supplementary file 1 — Supplementary Material 1 [file 12911_2023_2319_MOESM1_ESM.docx]

**Annex 1: Data Collection Guide**

**Political economy analysis of HMIS in Tanzania**

*Respondents are advised to answer all questions with examples where relevant, and skip the irrelevant questions based on their level (government or non-government).*

Research questions

1. Who are the key actors/ stakeholders influencing the design, adoption and investment in Health Management Information System (HMIS) in Tanzania?
   1. What role does each of the mentioned actor/stakeholder play within the HMIS ecosystem?
      1. Government stakeholders (Ministries – Ministry of Health -MOH, Ministry of Finance and Planning - MOFP, President's Office - Regional Administration and Local Government -PO-RALG, etc)?
         1. Probe on the Ministry of Health programs are: - Reproductive Health Program, National Aids Control Program, National Immunization Program, National TB/Leprosy Program, Neglected Tropical Disease Program, National Malaria Program, Pharmaceutical Unit [also Medical Store Department - MSD??]
2. Development partners and funders (World Bank/Global Fund/DFID / PEPFAR/President’s Malaria Initiative/GAVI / etc)?
3. Technical Agencies: WHO/UNICEF/UNDP/ UNAIDS /CHAI/UNAIDS/ PEPFAR/USAID/CDC/ etc)?
4. Implementing partners (e.g. NGOs including PATH, SIKIKA/AMREF)?
5. Sub-national level stakeholders?
   1. What factors influencing the decision to invest in HMIS in Tanzania?

i. Government priorities and interests?

ii. Development partners and funders priorities and interests?

iii. Technical Agencies priorities and interests?

iv. Implementing partners’ priorities and interests?

v. Availability of financial resources?

vi. Capacity in financial management/ resource coordination?

1. How is the relationship across actors/stakeholders in the decision-making process within HMIS ecosystem in Tanzania?
   1. How do ministries, departments and donors relate during meetings for decision making around HMIS?
      - Probe on how such relationship affects national and sub national level priorities?
   2. What is the relationship between donors who provides financial support for HMIS strengthening? How do they interact? Is it frequently?
      - Probe on how such relationship affects national and sub national level priorities?
   3. How do development/funding partners and technical agencies relate during meetings for decision making around HMIS?
      - Probe on how such relationship affects national and subnational level priorities?
   4. How do development/funding partners and implementing partners relate during meetings for decision making around HMIS?
      - Probe on how such relationship affects national and subnational level priorities?
   5. Which donors take part in the priority setting process for HMIS strengthening?
   6. Do decision to invest or design HMIS reached collectively involving multiple stakeholders? If yes how? If no, why?
2. Forms of power (1) power over; 2) power to; 3) power with, and; 4) power within). How the power is exercised and how this influences HMIS ecosystem in Tanzania?

*Donor level*

- - - 1. Who are the major funders supporting HMIS programme?
         1. How funding for HMIS is being coordinated?
         2. What are the financial commitments of each donor towards HMIS strengthening?
      2. What role and influence do development partners/ funders have within the HMIS ecosystem?
      3. In your opinion, which donors influence the HMIS financial resource allocation? How do they influence this?
      4. In your opinion, which donors influence the annual work planning process for HMIS? How do they influence this?
      5. Which donors are involved in the budgeting and planning process for HMIS? why? Who should not be involved? why?
      6. At what stage of the budgeting/annual work planning process are donors involved?
      7. Which donors do participate in decision making for HMIS strengthening?
         1. What is the role of each donor in the decision-making process for the HMIS?
      8. Which donors are (are not) quite influential/ whose voice is heard most in meetings?
      9. What is the relative influence of different donors in the decision-making process?
         1. Are there any incentives in the decision-making process? If yes, what are those incentives?
         2. How does access to crucial information on HMIS ecosystem influence decision making process?
         3. How does access to financial resources needed to support HMIS ecosystem influence decision making process?
         4. What is the relative influence of different donors in the decision-making process?
         5. Are there any incentives in the decision-making process? If yes, what are those incentives?

*Technical agencies*

- - - 1. What role and influence do technical agencies have within the HMIS ecosystem
      2. In your opinion, which technical agencies influence the annual work planning process for HMIS? How do they influence this?
      3. Which technical agencies do participate in decision making for HMIS strengthening?
      4. Which technical agencies are (are not) quite influential/ whose voice is heard most in meetings?
      5. What is the relative influence of different technical agencies in the decision-making process?
      6. Are there any incentives to different technical agencies in the decision-making process? If yes, what are those incentives?

*Implementing partners*

1. What role and influence do implementing partners have within the HMIS ecosystem?
2. In your opinion, which implementing partners influence the annual work planning process for HMIS? How do they influence this?
3. Which implementing partners do participate in decision making for HMIS strengthening?
4. Which implementing partners are (are not) quite influential/ whose voice is heard most in meetings?
5. What is the relative influence of different implementing partners in the decision-making process?
6. Are there any incentives to different implementing partners in the decision making process? If yes, what are those incentives?

*National level (ministries such as MoH, MoFP, PO-RALG etc.)*

- - - 1. In your opinion, who influences the HMIS financial resource allocation? How do they influence this?
         1. How funding for HMIS is being coordinated?
      2. In your opinion, who influences in the annual work planning process for HMIS? How do they influence this?
      3. Which departments within the ministries are involved in the budgeting and planning process for HMIS? why? Which should not be involved? why?
      4. At what stage of the budgeting/annual work planning process is each of the Ministries and Departments involved?
      5. What is the role of each Ministries and Departments in the decision-making process for the HMIS?
      6. What is the relationship between the Ministries and Departments you have mentioned? How do they related in coordination or when participating in meetings?
      7. What is the relative influence of different ministries and departments in the decision-making process?
         1. Influence in terms of financial resource mobilisation or coordination?
         2. Influence in terms of adopting certain HMIS design elements? (e.g., move towards reduced fragmentation)
         3. Influence in terms of coordinating views and resources from different stakeholders?
      8. How the power differences among actors do affects HMIS ecosystem? (Frustration, reduced motivation, incentives, suspicion, reduced trust etc.)
      9. What are the facilitating or limiting factors towards collective action for HMIS strengthening?

1. What are the main motives or interests among stakeholders/ actors regarding the design and adoption of HMIS?
   - - - 1. Think of the following actors:
     1. Donor who are funding the HMIS strengthening in Tanzania?
     2. Development partners who are supporting the HMIS strengthening in Tanzania?
     3. Technical agencies which are also supporting the HMIS strengthening in Tanzania.
     4. Government stakeholders (ministries) overseeing the HMIS strengthening in Tanzania?
     5. Implementing partners such as; Non-government organization (NGOs) stakeholders overseeing the HMIS strengthening in Tanzania?
        - 1. To what extent the interests of different stakeholders affect the decision-making regarding financing and/or designing towards HMIS strengthening?
          2. If available, what are the existing mechanisms to ensure harmonisation of power differences and stakeholders’ interests?
          3. If available, how the varied views from different stakeholders regarding HMIS are being coordinated for fair decision making towards designing and adoption of HMIS?
2. What should be done potentially to improve HMIS ecosystem in Tanzania?
3. What plausible areas for intervention have been identified to improve HMIS design, adoption and implementation?
4. Looking at the power differences among different actors (e.g., funders, technical agencies, implementing partners etc)?
5. Looking at the decision-making process? Who should be involved? How best to coordinate the decision-making process for actionable decisions*?*
6. Coordination of the resources? How best to coordinate financial support?
